# Incidental Monotypic (Fat-Poor) Renal Angiomyolipoma Diagnosed by Core Needle Biopsy

**DOI:** 10.1155/2012/906924

**Published:** 2012-04-03

**Authors:** Verena Kufer, Siegfried A. Schwab, Maike Büttner, Abbas Agaimy, Michael Uder, Kerstin Amann

**Affiliations:** ^1^Department of Pathology, University of Erlangen-Nürnberg, 91054 Erlangen, Germany; ^2^Department of Radiology, University of Erlangen-Nürnberg, 91054 Erlangen, Germany

## Abstract

We present the case of a 55-year-old patient with a history of chemotherapy and bone marrow transplantation because of acute myeloid leukaemia. An incidental 4 × 3 cm measuring renal mass was detected while performing a magnetic resonance imaging (MRI) for lumbago. The lesion was suspected to be either a renal cell carcinoma (RCC) or a leukemic infiltration. To decide about further treatment a percutaneous core needle biopsy was performed. Histology showed a monotypic angiomyolipoma, a relatively rare benign renal lesion. Interestingly, in cross-sectional imaging, angiomyolipoma was not taken into differential diagnostic account because of lack of a fatty component. Due to bleeding after biopsy the feeding artery of the tumor was occluded by microcoils. This case demonstrates the utility of biopsy of renal tumors, in particular when small tumor-like lesions are incidentally detected to decide about the right treatment and thereby avoiding nephrectomy.

## 1. Introduction

Due to advancements in modern imaging during the last years, nowadays more than 70% of kidney tumours are detected incidentally [1]. In most of the cases appropriate treatment can be initiated on the basis of cross-sectional imaging modalities without further investigations because of the mass showing certain signs of malignancy or coming up with published imaging criteria of benign lesions like cysts or fat-containing angiomyolipoma [2]. If it is not possible to make a reliable diagnosis, percutaneous biopsy should be taken into account to avoid unnecessary surgery.

The pretest probability of benign lesions is determined by tumor size: recent studies showed that up to 26.3% of small (≤4 cm) renal lesions and 16.8% of the masses >4 cm are benign [3]. Most frequently they are angiomyolipomas [4].

Angiomyolipoma belongs to the family of perivascular epitheloid cell tumors (PEComa). In their classical form, they present with a triphasic histology comprising variable proportions of smooth muscle cells, thick-walled tortuous blood vessels, and mature adipose tissue.

Immunohistochemically, the myogenic component shows a variable expression of smooth muscle markers (sm-actin, desmin or h-caldesmon) and melanocytic markers (HMB-45 and Melan-A) [5].

The mean age of patients with angiomyolipoma is about 50 years and 78% of them are females [6]. Most angiomyolipomas are sporadic and rarely they do occur as part of the tuberous sclerosis complex [7]. Both variants show alterations involving the TSC gene [8].

## 2. Case Report

Our patient, a 55-year-old woman, was diagnosed with acute myeloid leukaemia and treated by allogenic stem cell transplantation.

Developing lumbago a year later magnetic resonance imaging (MRI) of the lumbar spine was performed. Along with an inhomogeneous signal intensity of the vertebral bone marrow (which was attributed to the underlying acute myeloid leukaemia) and a spinal disc herniation at Th 8/9 without relevant spinal stenosis, a 4 × 3 cm mass lesion in the lower pole of the left kidney was seen. The patient had no stigmata of the tuberous sclerosis complex.

**Figure 1:**
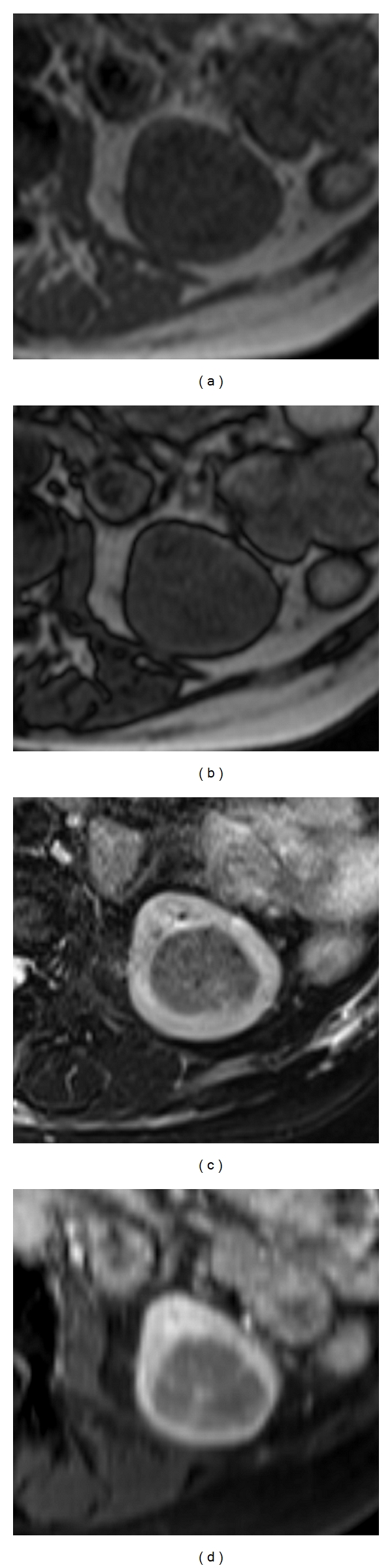
(a–d) Axial MR images of the left kidney, T1-weighted in-phase (a) T1-weighted opposed phase (b) T2-weighted fat-saturated (c), and T1-weighted, contrast-enhanced fat-saturated (d). The left renal mass is isointense in T1-weighted imaging without evidence of fatty components (a, b); the small hypointense spots in fat-saturated T2-weighted imaging are vessels (c). The mass is hypointense in T1-weighted imaging after i.v. contrast media (d); solley centrally there are KM-enhancing foci representing vessels (d).

In a subsequent MRI of the kidneys the lesion was classified as highly suspicious for RCC. As fatty tissue could not be identified, an angiomyolipoma was not included in the differential diagnosis (Figures 1(a)–1(d)).

It was decided to perform a percutaneous biopsy of the renal tumor under computerized tomography (CT) guidance. A 17G-coaxial biopsy needle was inserted into the tumor under local anesthesia and three core needle biopsies were taken.

**Figure 2:**
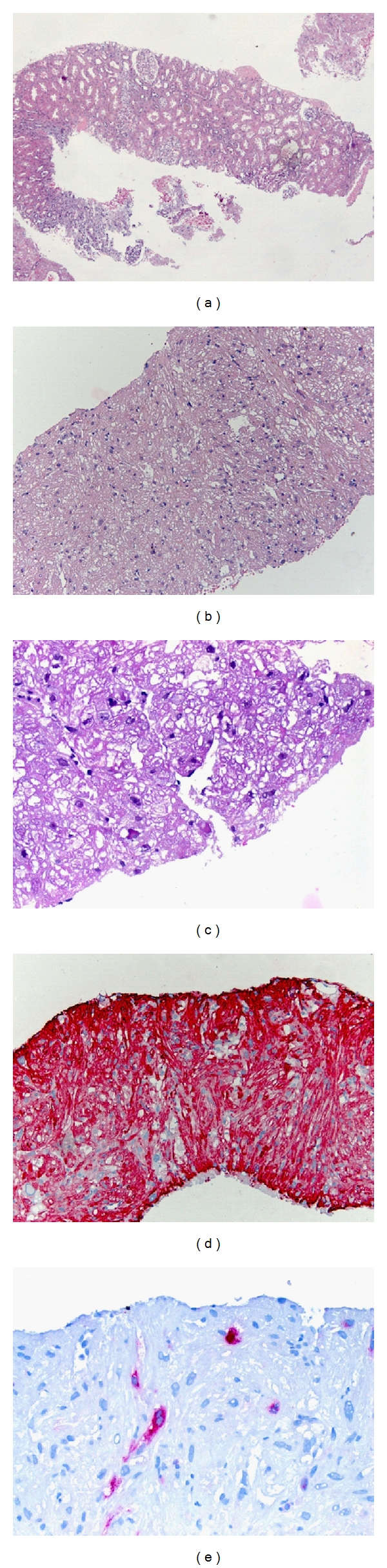
Representative findings of the kidney biopsy (a) with the angiomyolipoma (b–e). (a) Light microscopy of the kidney, hematoxylin stain (magnification ×4). (b) Light microscopy of the angiomyolipoma, hematoxylin stain (magnification ×100). (c) Higher magnification of the tumor showed large spindled and epithelioid cells with clear and eosinophilic cytoplasm intimately associated with slit-like vascular channels, (magnification ×400). (d) Immunohistochemistry using an antibody against actin (magnification ×100) shows a strong positivity of the smooth muscle component of the tumor. (e) Immunohistochemistry using an antibody against HMB-45 (magnification ×100) shows a few positive tumor cells.

On histology renal parenchyma from the cortex showed 8 glomeruli with a slight increase of mesangial cells and matrix and ectatic tubuli (Figure 2(a)). Within the renal tissue there were tumorous infiltrates composed of spindled and large polygonal (polymorphic) tumor cells with hyperchromatic and partially bizarre nuclei. The tumor cells were arranged in short irregular intersecting fascicles. The cytoplasm ranged from eosinophilic and granular to clear with well-recognizable cell borders. Only 1-2 mitoses/10 HPF could be detected. Infiltration of acute myeloid leukaemia could be excluded histologically (Figures 2(b)-2(c)).

Immunohistochemistry was performed on 3 *μ*m paraffin sections using a polymer Kit purchased from Zytomed systems Ltd., Berlin, Germany according to the manufacturer's instructions and Dab as a chromogen. Negative controls were used throughout. The renal tubular epithelial cells served as endogenous negative control. The tumor cells were strongly positive for smooth muscle actin (Figure 2(d)). Scattered large tumor cells showed a strong granular cytoplasmic positivity for HMB-45 (Figure 2(e)). The tumor cells were negative for pan-cytokeratin marker (KL-1), S100, renal cell carcinoma marker RCC, CD10, and CD34. The proliferation index assessed by MiB-1 was low (about 1%).

A control CT following biopsy procedure revealed a relevant perirenal hemorrhage with active extravasation of contrast media. Subsequently, the bleeding artery was occluded using microcoils. In the control angiography there was no remaining extravasation, and the bleeding vessel was entirely occluded. On last followup (25 months), the patient was alive without evidence of tumor progression or metastasis.

## 3. Discussion

The classic form of angiomyolipoma does not pose great diagnostic difficulties on cross-sectional imaging relying on the detection of macroscopic fat within a renal mass [9]. However, there are some angiomyolipomas that consist of over 95% of one component only, for example, adipose tissue (lipomatous variant) or more often smooth muscle cells (leiomyomatous variant) [6]. Tumors which contain less than 25% fat, as in the current case (so-called “fat-poor type”), present a differential diagnostic problem to radiologists so that most are suspected to be RCC on imaging.

Histological diagnosis of the classic variant of renal angiomyolipoma is usually straight forward. To differentiate angiomyolipoma from RCC sensitive and specific markers are established nowadays [5].

The differential diagnosis of monotypic renal angiomyolipoma includes true smooth muscle tumors and RCC on the one hand and liposarcoma on the other hand. The tumor in the current case had a dominant smooth muscle component that coexpressed both smooth muscle actin and HMB45. The coexpression of these two markers (myomelanocytic profile) is considered to be unique for angiomyolipoma and other members of the PEComa family, thus ruling out the possibility of RCC or liposarcoma. Furthermore, the tumor showed a negative staining for CD10, RCC, and KL-1, markers typically expressed in RCC. Particularly in tumors with more pronounced HMB45 expression and less prominent reactivity for myogenic markers, metastatic malignant melanoma represents an important consideration. Malignant melanoma may display cytomorphological similarity to monotypic epithelioid angiomyolipoma, but it usually shows features of frank malignancy and a more diffuse and strong expression of HMB45 and protein S100 and lack myogenic markers.

The nomenclature and biological assessment of renal angiomyolipoma have been controversial. While the presence of epithelioid areas within otherwise typical (triphasic) tumors is generally considered of no significance, evidence is accumulating that purely epithelioid renal angiomyolipoma (synonymously referred to as renal PEComa) may carry an increased risk for malignant behaviour. In a recent study, Nese et al. [10] analyzed a large series (*n* = 41) of well-characterized pure (monotypic) epithelioid renal angiomyolipomas (PEComas) for risk of tumor progression. They have characterized associated tuberous sclerosis complex, concurrent angiomyolipoma in the kidney or other organ, tumor necrosis, tumor size >7 cm, extrarenal extension and/or renal vein involvement, and carcinoma-like growth pattern as adverse clinicopathologic parameters associated with disease progression. While tumors with <2 adverse prognostic parameters as in our current case were considered to be of low risk for progression, those with ≥4 adverse prognostic parameters carried a high risk of progressive disease and most have recurred or metastasized. These findings underline the malignant potential of monotypic renal angiomyolipoma (PEComa) thus necessitating surgical excision of larger tumors that carries a significant risk. However, that series was biased by including mostly cases published previously or selected for the study from more than 40 centers and by excluding cases with bland-looking histology. Accordingly, most of analyzed cases had a remarkable or probably malignant histology. This is evident from a mean tumor size of 11.9 cm in that study which is very unusual for a majority of renal angiomyolipoma [10, 11].

A definite preoperative diagnosis of a renal tumor is pivotal due to the different treatment strategies of RCC and angiomyolipoma. While most small RCCs can be treated with partial nephrectomy, and those in higher surgical risk patients may be treated with less invasive modalities such as cryoablation or RFA, thereby preserving renal function as compared with radical nephrectomy for larger RCC, active surveillance may be performed for patients with asymptomatic angiomyolipoma, even in those with large tumors [12]. The treatment of symptomatic (usually flank pain) and complicated (retroperitoneal hemorrhage) angiomyolipoma comprises nephron-sparing surgery or selective arterial embolization for angiomyolipoma smaller than 4 cm [9, 13].

Several studies showed that tumor biopsy is an effective and safe approach to make a correct diagnosis of renal masses: the sensitivity of core needle biopsy is 70–100% with a specificity of 100% and an accuracy of more than 90% [14, 15].

It has been shown that the highest sensitivity and the highest negative predictive values (97% and 89%) are found in masses between 4 and 6 cm. They are lower in masses smaller than 3 cm (84% and 60%) and masses bigger than 6 cm (87% and 44%) [16].

Possible reasons for false negative biopsies are sampling errors due to small lesions, which are difficult to target or biopsies out of necrotic areas in larger tumors [14, 15]. Therefore exact biopsy technique has to be performed to reduce the risk of missing small lesions. Furthermore pre-bioptic imaging has to be thoroughly analysed to differentiate between vital and necrotic components of renal masses.

As illustrated by our case the risk of a post interventional haemorrhage has to be taken into account. Patients should remain under observation after the procedure and follow-up imaging should be performed if clinical symptoms of haemorrhage are noted. However according to the literature clinically significant bleeding nowadays is considered to be rare [15] and interventional radiologic occlusion of the bleeding vessel(s) may be used for acute treatment as shown in this case. Nevertheless it has to be mentioned that in case of a malignant diagnosis (e.g., RCC) in our particular patient the definitive treatment of the renal mass probably may have needed to wait for hematoma resolution.

Therefore, if a tumor is diagnosed with confidence on cross-sectional imaging there is no need for percutaneous biopsy. Biopsy should be performed, however, in difficult to classify renal masses, patients with extra renal primary tumors, renal masses that may be caused by infection, consideration for percutaneous ablation, and in unresectable patient with comorbidity [17].

In the present case we decided to perform CT-guided renal biopsy because the tumor was difficult to classify on cross-sectional imaging, the patient's history of leukaemia with the possibility of recurrence and renal mass caused by infection. Furthermore the tumor size of 4.0 cm holds a relatively high percent of benign masses and a high sensitivity and high specificity of renal biopsy. 

In conclusion, this case illustrates the utility of needle biopsy for evaluation of incidentally detected asymptomatic small renal tumors. CT-guided biopsy is an effective procedure to make an accurate diagnosis and facilitate planning of the appropriate treatment and can help to avoid more aggressive surgical approaches and save patients' kidneys.

## Abbreviations

CT: Computed tomography,
MRI: Magnetic resonance imaging,
RCC: Renal cell carcinoma,
RFA: Radio frequency ablation.
